# Standardized High-Resolution Ultrasound Protocol for the Diagnosis and Monitoring of Carpal Tunnel Syndrome: A Mixed-Design Observational Study

**DOI:** 10.3390/diagnostics15131593

**Published:** 2025-06-23

**Authors:** Fabiana Battaglia, Luigi Troisi, Emanuele Cigna, Francesco Stagno d’Alcontres, Vincenzo Rizzo, Gabriele Delia

**Affiliations:** 1 Department of Plastic and Reconstructive Surgery, University Hospital of Messina “AOU Gaetano Martino”, Via Consolare Valeria 1, 98124 Messina, Italy; fstagnodalcontres@unime.it (F.S.d.); gabriele.delia@unime.it (G.D.); 2 IRCCS MultiMedica Group, University Department of Hand Surgery and Rehabilitation, San Giuseppe Hospital, 20123 Milan, Italy; luigi.troisi@unimi.it; 3 Plastic Surgery and Microsurgery Unit, Department of Translational Research and New Technologies in Medicine and Surgery, University of Pisa, 56122 Pisa, Italy; emanuele.cigna@gmail.com; 4 Department of Clinical and Experimental Medicine, University of Messina, 98125 Messina, Italy; v.rizzo@unime.it

**Keywords:** carpal tunnel syndrome, ultrasonography, median nerve, cross-sectional area, non-invasive diagnosis, nerve decompression

## Abstract

**Background/Objectives**: Carpal tunnel syndrome (CTS) is a common entrapment neuropathy. Traditional diagnostics like EMG and NCSs are invasive and do not visualize nerve morphology. This study aims to evaluate the diagnostic and prognostic value of high-resolution ultrasonography in patients with CTS using a standardized scanning protocol and to evaluate the relationship between sonographic findings and traditional electrodiagnostic results. **Methods**: In this observational study with both prospective and retrospective components, 31 subjects were included. Between November 2023 and June 2024, 11 symptomatic CTS patients were scheduled for surgical decompression and 14 healthy controls were prospectively enrolled. Additionally, six post-surgical CTS patients who had undergone decompression between 2016 and 2021 were retrospectively included for comparative analysis. All underwent clinical and ultrasonographic assessments of the median nerve at predefined anatomical landmarks. EMG was performed in the CTS groups. Ultrasound was repeated at 1, 3, and 6 months postoperatively to monitor morphological changes. **Results**: CTS patients had significantly increased the median nerve CSA compared to controls. Postoperative ultrasound showed progressive CSA reduction correlating with clinical improvement and EMG recovery. The CSA correlated moderately to strongly with distal motor latency. **Conclusions**: High-resolution ultrasound is a reliable, non-invasive tool for diagnosing and monitoring CTS. Standardized protocols are needed to support broader clinical adoption and establish it as a standalone diagnostic method.

## 1. Introduction

Carpal tunnel syndrome (CTS) is a prevalent entrapment neuropathy, affecting a significant portion of the adult population, particularly women, with peak incidence occurring between 40 and 60 years of age [1,2]. Despite its high frequency, diagnostic techniques for CTS remain limited in terms of non-invasive and completely painless options. Traditionally, the diagnosis relies on clinical symptoms, including paresthesia, nocturnal pain, and positive Tinel’s and Phalen’s signs, supported by electrodiagnostic studies such as electromyography (EMG) and nerve conduction studies (NCSs) [3,4]. However, these tests, while highly specific and sensitive, are invasive, uncomfortable for the patient, time consuming, and fail to provide direct visualization of nerve morphology [5].

Recent advancements in ultrasonography (US) have introduced an alternative diagnostic approach that is non-invasive, repeatable, cost-effective, and well-tolerated by patients [6]. Thanks to technological advancements such as high-frequency linear probes, improved axial resolution, and Doppler sensitivity, ultrasonography has become increasingly precise in evaluating peripheral nerves, allowing for a detailed structural and vascular assessment of the median nerve. High-resolution ultrasonography allows real-time imaging of the median nerve, offering quantitative measurements such as the cross-sectional area (CSA), nerve flattening ratio, and vascularity assessment through Doppler techniques [7,8]. Furthermore, ultrasonography has been recognized as a valuable tool for postoperative follow-up, providing insight into nerve recovery and potential complications such as fibrosis or incomplete release of the transverse carpal ligament [9].

Despite these advantages, ultrasonography has not yet been established as a gold standard for CTS diagnosis due to a lack of universally accepted diagnostic criteria. The threshold values for the CSA remain inconsistent across studies, with proposed cutoffs ranging from 6.5 mm^2^ to over 13 mm^2^, leading to variability in sensitivity and specificity [3,10,11]. While some authors consider electrophysiological tests as the primary reference standard, others incorporate clinical presentation as a determining factor, further complicating standardization efforts [6,12,13].

Based on these premises, we hypothesized that a standardized ultrasound protocol could reliably detect morphological changes in the median nerve consistent with CTS severity and that these changes would correlate with both clinical symptoms and electrodiagnostic findings.

This study aims to evaluate the diagnostic and prognostic role of ultrasonography in CTS patients by assessing preoperative and postoperative nerve morphology using a standardized, reproducible imaging protocol based on anatomical surface landmarks and comparing these findings with traditional electrodiagnostic tests. Specifically, our objectives include the following:

- Demonstrating the utility of ultrasonography as a non-invasive, painless, and reproducible technique for diagnosing CTS.
- Comparing ultrasonographic findings with electromyography/electroneurography to assess their correlation and potential complementary use.

By systematically analyzing the recovery of nerve trophism and functionality, this research seeks to contribute to the growing evidence supporting the integration of ultrasonography into CTS diagnosis and postoperative monitoring. Establishing standardized ultrasonographic parameters may ultimately enhance the accuracy and accessibility of CTS diagnostics in clinical practice.

## 2. Materials and Methods

This study included both prospective and retrospective data. The prospective component was conducted at the Plastic Surgery Unit of Policlinico G. Martino in Messina between November 2023 and June 2024 and involved symptomatic CTS patients and healthy controls. The retrospective component consisted of data from patients previously operated on for CTS between 2016 and 2021. Informed consent was obtained from all participants prior to enrollment.

No formal sample size calculation was performed, as this was a pilot prospective observational study. Patients were consecutively enrolled over the study period to evaluate the feasibility and reproducibility of the proposed ultrasound protocol.

Patients were divided into three groups. Group A comprised 11 patients (7 females and 4 males) with clinically and electrophysiologically confirmed carpal tunnel syndrome (CTS) who underwent standard mini-open technique.

Group A underwent surgical decompression using the mini-open technique, which involves a small longitudinal incision over the carpal tunnel, division of the transverse carpal ligament under direct vision, and minimal soft tissue dissection to reduce recovery time and complications [10].

Group B included 6 patients who had undergone mini-open carpal tunnel release during the period 2016–2021 and whose age range was similar to Group A. The surgical technique used was consistent across both groups and involved a limited palmar incision for direct decompression of the transverse carpal ligament. These patients, although retrospectively selected, were reevaluated using the same ultrasonographic protocol and served as a post-surgical reference group to support interpretation of the recovery pattern observed in Group A. All patients in Group B were asymptomatic at the time of re-evaluation, reporting complete resolution of preoperative symptoms. Clinical examination and structured interviews confirmed the absence of residual sensory or motor deficits. Their inclusion served to provide a reference for long-term post-surgical recovery under optimal conditions. These patients were recalled between November 2023 and June 2024 and underwent a single standardized ultrasound evaluation as part of this study. Group C consisted of 14 healthy individuals (7 females and 7 males) with no clinical symptoms and no evidence of CTS on physical examination or nerve conduction studies. Demographic data, including age, sex, and the side of hand involvement, were recorded at the time of enrollment (Table 1).

Patients meeting the following inclusion criteria were enrolled: for Groups A and B, a positive history of typical CTS symptoms (dysesthesia, paresthesia, pain, burning, and/or motor deficit) associated with a positive Tinel’s and/or Phalen’s test, confirmed by electrophysiological study. Patients in Group C were required to have no history of compressive neuropathies, peripheral neuropathy, or previous hand surgery. Subjects with hand arthropathies or other systemic diseases (e.g., diabetes mellitus, rheumatoid arthritis) were excluded.

After enrollment, all patients underwent a comprehensive clinical evaluation. A detailed anamnesis was obtained using a structured questionnaire that captured demographic information, smoking habits, and associated comorbidities. An objective examination was then performed, which included clinical tests (Tinel’s and Phalen’s tests) to assess symptom severity. For instance, in Group A, the frequency of positive findings for each clinical sign was recorded (Table 2).

High-resolution ultrasound examinations were performed on all participants using the Esaote MyLa^TM^ (Esaote S.p.A., Genoa, Italy) One system equipped with a 25 cm linear-array transducer (SL3235, 18–6 MHz frequency range, axial resolution < 0.2 mm), enabling high-resolution imaging of peripheral nerves. All subjects were examined in a standardized position (seated with elbow flexed at 90°, palm up), using pre-warmed gel and minimal probe pressure to avoid nerve compression. Each measurement was repeated three times, and the average was recorded to improve reproducibility.

All ultrasound examinations were performed by a single experienced radiologist with over 5 years of expertise in musculoskeletal imaging, thereby reducing interobserver variability.

All ultrasound scans were performed using a uniform preset. Depth was set between 3 and 4 cm to center the nerve in the field of view; gain was kept between 55% and 60%; focus was placed at the nerve level. The same ultrasound system was used for all patients, and settings were adjusted minimally when necessary to optimize image quality while maintaining protocol consistency.

Further details on probe positioning, measurement landmarks, and acquisition protocol are provided in Supplementary File S1.

To reduce observational bias, the ultrasonographer was blinded to both clinical diagnosis and EMG results at the time of the ultrasound assessment. Likewise, the EMG operator was blinded to the ultrasound findings.

The cross-sectional area (CSA) of the median nerve was measured at two standardized anatomical levels:

- The proximal forearm, corresponding to 2 cm proximal to the pisiform bone (before the nerve enters the carpal tunnel).
- The tunnel inlet, at the level of the pisiform bone.

At 3 cm distal to the pisiform (tunnel outlet), CSA was not measured due to anatomical variability and technical limitations related to nerve branching. In this distal segment, only vertical thickness was recorded as a complementary morphometric parameter.

**Figure 1:**
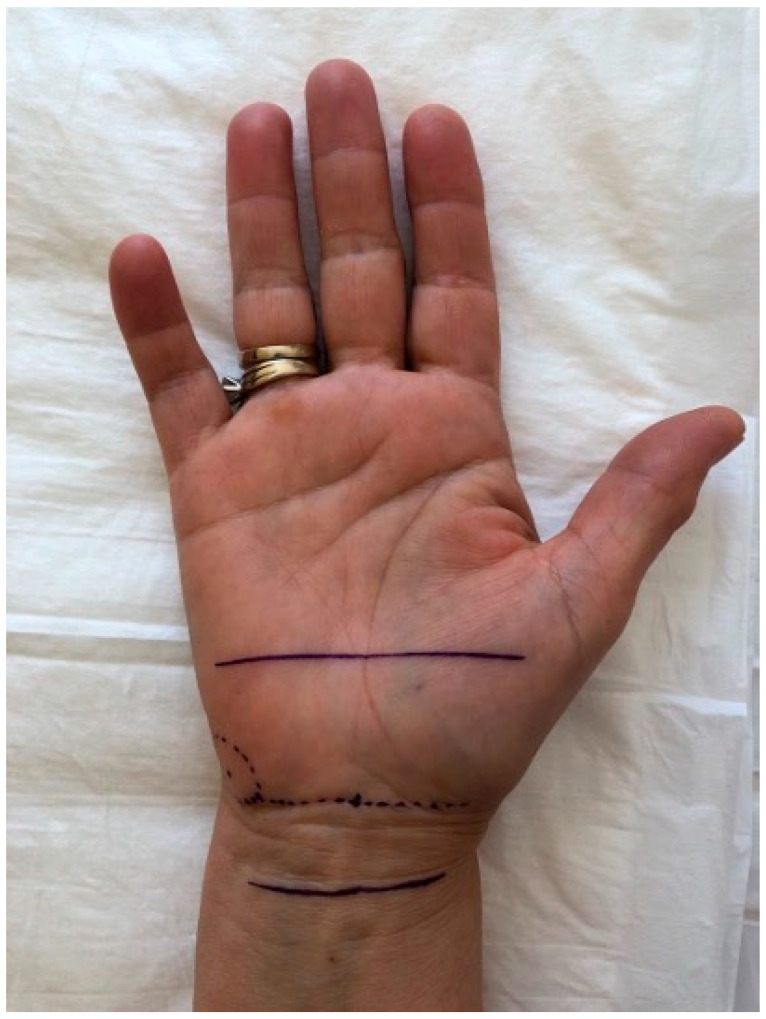
Surface anatomical landmarks for standardized sonographic evaluation of the median nerve in carpal tunnel syndrome. The central line (P) indicates the level of the pisiform bone, which corresponds to the carpal tunnel inlet. The proximal line (P−2 cm) is located 2 cm above the pisiform and represents the forearm level. The distal line (P+3 cm) is located 3 cm below the pisiform and corresponds to the mid-carpal tunnel level. These landmarks assist in consistent probe placement and allow reproducible cross-sectional area measurements of the median nerve along its course.

To enhance reproducibility, all ultrasound scans were performed with reference to anatomical surface landmarks clearly mapped on the palm, as illustrated in Figure 1. The pisiform bone was identified as the central reference landmark (0 cm), and standardized measurement points were determined along a straight longitudinal axis drawn on the volar forearm and palm. The “2 cm proximal” and “3 cm distal” positions were consistently marked using a flexible ruler from the pisiform, regardless of wrist size. This approach ensured uniform probe positioning and allowed reproducible CSA and thickness measurements across all patients. The ultrasound probe was oriented perpendicularly to the longitudinal axis of the forearm to obtain standardized transverse views of the median nerve. Minimal transducer pressure was applied to avoid nerve compression, using a generous amount of ultrasound gel to optimize acoustic coupling. All measurements were taken with the patient seated, elbow flexed at 90°, and palm supinated, to standardize positioning and ensure reproducibility. A consistent insonation angle of approximately 90° was maintained throughout all examinations to avoid anisotropy and ensure reliable cross-sectional imaging of the median nerve.

Preoperative ultrasound data for Group A are shown in Table 3.

For patients with CTS (Groups A and B), preoperative electromyography (EMG) was performed by an experienced technician under the supervision of a neurologist using standard protocols. The EMG study evaluated both motor and sensory components of the median nerve. Specifically, the protocol included stimulation of the median nerve at the wrist and elbow, with recording from the abductor pollicis brevis muscle for motor responses. For sensory conduction studies, antidromic stimulation was used with ring electrodes placed on the second digit. Standard filter settings were 20 Hz to 10 kHz for motor studies and 20 Hz to 2 kHz for sensory studies. Normal reference values were defined as follows: for motor fibers, a distal motor latency (DML) of less than 4 ms, a motor action potential (MAP) amplitude greater than 4.5 mV, and a conduction velocity greater than 50 m/s; for sensory fibers, a sensory nerve action potential (SNAP) amplitude greater than 10 µV and a sensory conduction velocity greater than 50 m/s. Preoperative EMG data for Group A are summarized in Table 4, and those for Group B in Table 5.

For Group A, postoperative ultrasound examinations were performed at 1, 3, and 6 months after surgery to assess the evolution of the median nerve morphology. Each examination was carried out using the same standardized scanning protocol. For example, at 1 month postoperatively, changes in the cross-sectional area and other parameters were recorded (see Table 6).

Similarly, at 3 and 6 months postoperatively, further improvements were observed. The ultrasound parameters gradually normalized, with CSA at the pisiform level decreasing to 9.0 ± 2.0 mm^2^ at 3 months and to 8.5 ± 1.8 mm^2^ at 6 months (Table 7 and Table 8, respectively). These values closely approached those observed in the control group (8.0 ± 1.5 mm^2^), as reported in Table 9.

For the control group (Group C), ultrasound examination was performed only once. The median nerve parameters in healthy subjects were within the normal range (Table 9).

### Statistical Analysis

All statistical analyses were performed using SPSS (Version 25) and GraphPad Prism (Version 8). Continuous variables are expressed as mean ± standard deviation (SD), while categorical variables are reported as frequencies (percentages). The normality of the data was assessed using the Shapiro–Wilk test.

For normally distributed continuous variables, comparisons between groups were conducted using Student’s *t*-test (for two-group comparisons) or one-way analysis of variance (ANOVA) with Bonferroni post hoc correction (for comparisons among more than two groups). The assumption of homogeneity of variances was tested using Levene’s test prior to conducting ANOVA. Only comparisons meeting both normality and variance homogeneity assumptions were analyzed with ANOVA and Bonferroni post hoc tests. When data were not normally distributed, the Mann–Whitney U test or the Kruskal–Wallis test was applied. The choice of statistical tests for each comparison was guided by the distribution of the data as assessed by the Shapiro–Wilk test. Parametric tests (*t*-test, ANOVA) were applied when assumptions of normality were met, while non-parametric alternatives (Mann–Whitney U, Kruskal–Wallis) were used for skewed distributions. Categorical variables were analyzed using the chi-square test or Fisher’s exact test, as appropriate.

For Group A, where ultrasound parameters were measured at multiple time points (preoperative, 1, 3, and 6 months postoperatively), repeated measures ANOVA was used to assess temporal changes. Post hoc pairwise comparisons were performed if the overall test reached statistical significance.

Pearson’s correlation coefficient was used to evaluate the relationship between ultrasound parameters and electromyography (EMG) values when the data were normally distributed; otherwise, Spearman’s rank correlation was applied.

A *p*-value of <0.05 was considered statistically significant.

Correlation analyses were performed between sonographic parameters (CSA at the pisiform level, CSA at 2 cm proximal, and nerve thickness) and neurophysiological measures, including distal motor latency (DML), motor action potential amplitude (MAP), sensory conduction velocity, and symptom severity scores. Spearman’s rho was used due to the ordinal nature and non-normal distribution of some variables.

Comparison of preoperative ultrasound parameters among Groups A (CTS patients), B (previously operated CTS patients), and C (control group) (Table 10).

## 3. Results

A total of 31 subjects were enrolled in this study. The demographic characteristics are summarized in Table 1.

A one-way ANOVA was performed to compare the mean age across the three groups, revealing a statistically significant difference (*p* = 0.034). Post hoc analysis with Bonferroni correction indicated that Group A (mean age 64.1 ± 12.1 years) was significantly older than Group B (mean age 53.5 ± 9.2 years, *p* = 0.041), while no significant differences were found between Group B and Group C or between Group A and Group C.

In Group A, the 11 patients had a mean age of 64.09 ± 12.09 years, with right-hand involvement in 54.5% of cases, left-hand in 36.4%, and bilateral symptoms in 9.1%. Group B (*n* = 6) had a slightly lower mean age (53.5 ± 9.18 years) with a higher percentage of bilateral involvement (33.3%). The control group (Group C) had a balanced distribution of hand dominance and an average age of 54.0 ± 7.56 years.

Clinically, all patients in Group A presented with typical CTS symptoms. As shown in Table 2, 100% of patients (11/11) reported dysesthesia, paresthesia, and pain. In addition, 63.6% (7/11) tested positive on Tinel’s test and 100% (11/11) on Phalen’s test, confirming the clinical diagnosis of CTS. These findings confirmed the clinical diagnosis and justified further electrophysiological and ultrasonographic evaluation.

Preoperative ultrasound measurements (Table 3) revealed that patients with CTS (Group A) had a significantly enlarged median nerve CSA at 2 cm proximal to the pisiform (13.0 ± 3.0 mm^2^) and at the pisiform level (12.0 ± 3.0 mm^2^) compared to values observed in healthy controls (Group C: 8.5 ± 1.5 mm^2^ and 8.0 ± 1.5 mm^2^, respectively), as shown in Table 10 (*p* < 0.001 for both levels). Similarly, the preoperative EMG data in Group A (Table 4) and Group B (Table 5) demonstrated altered conduction velocities and latencies, consistent with median nerve compression.

Postoperative follow-up in Group A showed progressive improvement in the ultrasonographic parameters. At 1 month postoperatively, the CSA at the pisiform level significantly decreased from 12.0 ± 3.0 mm^2^ to 10.0 ± 2.5 mm^2^ (*p* = 0.018). This reduction continued at 3 months (9.0 ± 2.1 mm^2^; *p* = 0.006 compared to baseline) and at 6 months (8.5 ± 1.9 mm^2^; *p* = 0.001 compared to baseline). Pairwise comparisons confirmed significant differences between baseline and each follow-up, while the change between 3 and 6 months was not statistically significant (*p* = 0.072), suggesting morphological stabilization. A similar trend was observed in nerve thickness at the pisiform level.

The pisiform level was selected for longitudinal assessment, as the CSA at this level is the most sensitive parameter for detecting compression and monitoring postoperative changes. Thickness was also included as a secondary morphological descriptor to complement the CSA and provide additional detail regarding nerve flattening over time.

The reduction in the CSA observed in Group A after surgery was not only statistically significant but also clinically relevant. Prior studies have suggested that a CSA decrease of more than 2 mm^2^ at the pisiform level may reflect effective decompression and symptom improvement. In our cohort, the average CSA decreased by over 3 mm^2^ within the first 3 months postoperatively, supporting the utility of sonographic monitoring. However, standardized thresholds for clinically meaningful change are not yet universally established, and further research is needed to define reference values applicable in routine care.

Our findings are consistent with previous studies demonstrating increased CSA values in patients with CTS compared to healthy controls, and a progressive CSA reduction after surgical decompression. For instance, recent studies have reported similar CSA thresholds at the pisiform level, typically ranging from 10 to 12 mm^2^, which are in line with the preoperative values observed in our Group A cohort. These findings are consistent with those reported by Wolny et al. [14,15] and Ikumi et al., who demonstrated that CSA measurements within this range are indicative of carpal tunnel syndrome severity. In terms of postoperative monitoring, our observation of a ≥3 mm^2^ CSA reduction within 3 months aligns with the results of early morphologic improvements on ultrasound, which were associated with favorable clinical recovery [16]. These parallels reinforce the reliability and prognostic value of ultrasound in both the diagnosis and follow-up of CTS, supporting its integration into routine clinical assessment. Changes over time in the CSA and thickness at the pisiform level are presented in Table 11.

In contrast, the control group (Group C) exhibited stable and normal median nerve measurements on ultrasound, as detailed in Table 9. These data reinforce the value of ultrasound as a reliable, noninvasive tool for diagnosing CTS and monitoring postoperative recovery.

The inclusion of Group B provided a long-term morphological reference for post-surgical outcomes, offering a valuable comparison for interpreting the recovery trajectory observed in Group A. The sonographic normalization observed in Group B supports the notion that CSA reduction and nerve reshaping are reliable indicators of successful decompression. This reinforces the use of standardized ultrasound protocols not only for diagnosis but also for monitoring and outcome validation in CTS management.

Overall, the combined clinical, electrophysiological, and ultrasonographic findings support the efficacy of surgical decompression in improving median nerve morphology and function.

Importantly, the normalization of sonographic parameters was significantly correlated with improvements in both distal motor latency (ρ = 0.86, *p* = 0.0008) and symptom severity scores (ρ = 0.80, *p* = 0.0031), reinforcing the clinical relevance of ultrasonographic follow-up.

## 4. Discussion

The use of ultrasound in diagnosing carpal tunnel syndrome (CTS) has evolved significantly since its introduction in the second half of the 20th century. The first studies demonstrating the diagnostic accuracy of ultrasound in CTS were conducted by Buchberger and colleagues, who established its potential in visualizing the carpal tunnel [6]. These findings were later validated by Altinok et al., who compared ultrasound with nerve conduction studies (NCSs), a widely accepted reference standard in CTS diagnosis [17].

A major breakthrough in ultrasound diagnostics was the introduction of the median nerve cross-sectional area (CSA) as a critical parameter. Duncan et al. were among the first to highlight its importance [18], and their findings were further corroborated by Lee et al., [19] Wong et al., [20] and Keleş et al. [21]. Additionally, Swen et al. identified the pisiform bone as a reliable landmark for CSA measurement, ensuring consistency across studies [22].

Our study confirms that a three-point linear scanning protocol using surface anatomical references (2 cm proximal, at pisiform, and 3 cm distal) allows consistent probe placement and facilitates reproducible measurements. This approach may contribute to establishing standardized acquisition methods across different centers.

Despite advancements in ultrasonographic diagnostics, standardized cutoff values for the median nerve cross-sectional area (CSA) remain inconsistent. Recent studies have reported thresholds ranging from 8.5 mm^2^ to 12.5 mm^2^, contributing to variations in diagnostic sensitivity and specificity [23,24]. Research by Aggarwal et al. suggests that a CSA threshold of 9 mm^2^ achieves high diagnostic accuracy, with 94% sensitivity and 100% specificity, making it one of the most reliable ultrasonographic parameters for CTS diagnosis [2].

While NCS remains the gold standard, it has several limitations, including its invasive nature, patient discomfort, high cost, and inability to provide direct morphological assessment of the median nerve [25]. In contrast, high-resolution ultrasonography offers a non-invasive, cost-effective, and real-time imaging technique that allows direct visualization of the nerve and surrounding structures. It has demonstrated comparable sensitivity and specificity to NCS in several studies [4]. Additionally, ultrasound can identify secondary causes of CTS, such as space-occupying lesions, synovial hypertrophy, and tenosynovitis, which may not be evident on NCSs [6,13].

Moreover, advanced ultrasonographic techniques, such as Color and Power Doppler, have been introduced to assess inflammatory changes and hypervascularization of the median nerve, contributing further to the understanding of CTS pathophysiology [26]. Some researchers have also explored additional parameters, such as the flattening ratio (FR), median nerve attenuation ratio, and palmar retinacular bowing, but their diagnostic utility remains less consistent compared to the CSA [4,27].

Various studies have attempted to classify CTS severity based on CSA measurements, correlating them with clinical and electrophysiological findings. The most commonly adopted classification includes the following:

- Mild CTS: CSA between 10 and 12.9 mm^2^.
- Moderate CTS: CSA between 13 and 14.9 mm^2^.
- Severe CTS: CSA ≥ 15 mm^2^ [28].

In our study, all patients in Group A presented with characteristic CTS symptoms, including 100% reporting dysesthesia, paresthesia, and pain, while 54.5% exhibited motor deficits. On physical examination, 63.6% of patients had a positive Tinel’s sign, and 100% had a positive Phalen’s test, supporting the clinical diagnosis.

The CSA at the pisiform level showed a strong positive correlation with both neurophysiological and clinical severity parameters. Specifically, the CSA correlated with distal motor latency (ρ = 0.86, *p* = 0.0008) and with the symptom severity score (ρ = 0.80, *p* = 0.0031). Furthermore, serial postoperative ultrasound examinations revealed a gradual decrease in CSA values over time, paralleling symptom improvement.

The debate on whether ultrasonography can replace NCSs as the primary diagnostic tool for CTS continues. Some researchers propose a diagnostic algorithm where ultrasound serves as the first-line test, with NCS reserved for non-diagnostic cases [20,29]. Our study supports this approach, reinforcing the role of ultrasonography as an effective, non-invasive, and accessible screening tool for CTS diagnosis.

Additionally, ultrasound plays a crucial role in treatment planning and follow-up. Steroid injections, commonly used for mild and moderate CTS, achieve better outcomes when guided by ultrasound, improving accuracy and reducing complications [30]. For severe CTS cases requiring surgical decompression, ultrasound helps confirm diagnosis and assess postoperative nerve recovery [31].

Despite its advantages, ultrasonography remains operator-dependent, requiring experience for reliable measurements. Additionally, variability in CSA cutoff values across studies highlights the need for standardized protocols to enhance reproducibility and diagnostic accuracy [13].

This study presents some limitations. First, ultrasonography is an operator-dependent technique, and although all scans were performed by an experienced examiner using a standardized protocol, some degree of subjectivity may still exist. Second, the relatively small sample size, particularly in Group A (*n* = 11) and Group B (*n* = 6), limits the statistical power of the analyses and may affect the generalizability of our findings.

In addition to the limited sample size, this study is potentially subject to selection bias, as participants were recruited from a single institution and may not fully represent the broader population of patients with CTS.

Additionally, although Group B was retrospectively identified based on prior surgical history, the ultrasound evaluations were conducted prospectively during the study period using the same standardized protocol as for Groups A and C, ensuring methodological consistency.

Although efforts were made to blind the ultrasound and EMG assessors to each other’s findings, the complete blinding of patients and investigators was not feasible in this observational design. This may have introduced some observational or expectation bias, despite standardized measurement protocols.

Nonetheless, the consistency of trends observed across multiple parameters and time points supports the internal validity of our observations and provides a foundation for future research involving larger cohorts.

Furthermore, the relatively small sample size, especially in Groups A and B, inherently limits the statistical power of the analyses. While several findings reached statistical significance, smaller effects may not have been detected. These results should therefore be interpreted with caution and confirmed in larger studies designed with adequate power.

In addition, the difference in age between groups, particularly between Groups A and B, may represent a potential confounding factor, as age could influence both nerve morphology and recovery patterns. Although statistical adjustment was not performed due to sample size, this aspect should be considered when interpreting longitudinal outcomes.

Another limitation, which reflects a broader issue in the field, is the absence of a standardized and universally accepted CSA cutoff value for the diagnosis of CTS. Different studies have proposed varying thresholds depending on patient populations, equipment, and measurement techniques. This variability limits inter-study comparability and complicates the clinical adoption of sonographic criteria. Establishing a consensus on diagnostic cutoffs should be a key focus of future multicenter research.

Regarding the study design, it is important to clarify that, while Groups A and C were prospectively enrolled, Group B included patients who had previously undergone CTS surgery between 2016 and 2021. These patients were retrospectively identified but were recontacted and re-evaluated during the study period (November 2023–June 2024) using the same standardized ultrasonographic protocol. Therefore, the sonographic data for all three groups were collected prospectively, ensuring methodological consistency in the imaging assessment. The retrospective nature of the surgical history for Group B does not affect the prospective collection of ultrasound measurements, which remain valid for comparative purposes within the study framework.

Moreover, recent developments in artificial intelligence and automated image analysis hold promise for integrating CSA recognition and classification algorithms into routine practice, potentially reducing operator dependency and enhancing diagnostic efficiency.

Future research should focus on establishing uniform ultrasonographic criteria and validating them through large-scale, multicenter studies involving diverse populations and clinical settings. These efforts are essential to improve diagnostic standardization and promote the global adoption of ultrasound as a reliable tool for the diagnosis and monitoring of CTS.

Our findings, along with previous studies, support the growing role of ultrasonography in CTS diagnosis and management. As technology advances, it is likely that ultrasonography will become a first-line, stand-alone diagnostic tool, offering a safer and more patient-friendly alternative to traditional electrophysiological testing. With continued research and validation, ultrasonography has the potential to revolutionize CTS diagnostics and improve clinical outcomes worldwide.

The present findings reinforce the role of high-resolution ultrasonography as a non-invasive and repeatable imaging modality for both diagnosis and postoperative follow-up in carpal tunnel syndrome. Sonographic parameters such as the CSA correlated with clinical severity and showed measurable improvement following decompression. However, the broader adoption of ultrasonography in clinical settings remains limited by the absence of standardized diagnostic cut-offs and reference values. This variability affects inter-study comparability and underlines the need for future multicenter research aimed at establishing consensus protocols.

Methodologically, although Group B included patients who had undergone CTS surgery prior to the study period, all ultrasound assessments were conducted prospectively using the same standardized protocol applied to Groups A and C. This approach ensured consistency in data collection and preserved the integrity of comparisons across cohorts.

## 5. Conclusions

In this prospective study, surgical decompression for carpal tunnel syndrome resulted in a progressive normalization of key sonographic parameters, particularly CSA and nerve thickness. These morphological changes correlated significantly with improvements in clinical symptoms and distal motor latency, supporting the efficacy of the surgical intervention. Ultrasonography emerged as a useful, non-invasive complementary tool in the assessment of postoperative recovery and nerve morphology.

## Figures and Tables

**Table 1:** Demographic characteristics and hand involvement.

Group	*n*	Females/Males	Mean Age (Years ± SD)	Hand Involvement (Right/Left/Both, %)
A	11	7/4	64.09 ± 12.09	Right: 54.5%, Left: 36.4%, Both: 9.1%
B	6	5/1	53.5 ± 9.18	Right: 50.0%, Left: 16.7%, Both: 33.3%
C	14	7/7	54.0 ± 7.56	Right: 50.0%, Left: 50.0% (one hand each)

**Table 2:** Clinical evaluation in Group A.

Patient	Dysesthesia	Paresthesia	Pain	Burning	Motor Deficit	Tinel	Phalen
R.B.	Yes	Yes	Yes	Yes	No	Yes	Yes
C.C.	Yes	Yes	Yes	No	No	No	Yes
M.C.	Yes	Yes	Yes	No	Yes	No	Yes
L.E.D.	Yes	Yes	Yes	No	Yes	No	Yes
G.I.	Yes	Yes	Yes	No	Yes	Yes	Yes
C.L.	Yes	Yes	Yes	No	Yes	No	Yes
D.M.	Yes	Yes	Yes	No	No	Yes	Yes
G.M.	Yes	Yes	Yes	Yes	Yes	Yes	Yes
C.S.	Yes	Yes	Yes	Yes	No	Yes	Yes
F.V.	Yes	Yes	Yes	No	Yes	Yes	Yes
C.Z.	Yes	Yes	Yes	No	No	Yes	Yes

**Table 3:** Preoperative ultrasound measurements (Group A).

Patient	Hand	Area at 2 cm Proximal (mm^2^)	Width at 2 cm Proximal (mm)	Thickness at 2 cm Proximal (mm)	Area at Pisiform (mm^2^)	Width at Pisiform (mm)	Thickness at Pisiform (mm)	Thickness at 3 cm Distal (mm)
R.B.	R	17.8	5.7	3.6	8.8	5.9	1.7	2.5
C.C.	L	13.6	6.8	3.0	6.8	5.1	2.1	2.0
M.C.	R	12.8	7.8	2.0	5.45	4.0	1.8	1.7
L.E.D.	L	12.5	3.4	3.3	14.0	7.3	1.6	1.4
G.I.	L	11.8	6.3	2.4	11.7	6.3	2.3	3.7
C.L.	R	9.8	5.7	2.3	14.4	6.9	2.5	2.0
D.M.	L	14.0	6.0	3.8	12.8	5.7	3.0	2.9
G.M.	R	17.8	5.7	3.8	8.7	6.0	1.7	2.5
C.S.	R	10.0	6.0	2.2	12.0	8.8	2.0	1.2
F.V.	L	14.0	6.0	3.7	12.7	5.5	3.0	2.5
C.Z.	R	8.4	4.4	2.8	6.5	5.0	1.8	1.5

Note: CSA is measured only at proximal and pisiform levels. At 3 cm distal, only vertical thickness (in mm) is recorded due to limited reproducibility of CSA at this level. Abbreviations: R (right) L (left).

**Table 4:** Preoperative EMG measurements (Group A).

Patient	Hand	VCS (m/s)	AMP (µV)	LDS (ms)	VCM (m/s)	AMP2 (mV)	LDM (ms)
R.B.	R	51	8	5.7	50	4	6.0
C.C.	L	47	9	5.2	40	10	6.8
M.C.	R	53	47	3.8	50	17	4.1
L.E.D.	L	54	7	4.3	52	5	5.6
G.I.	L	65	21	5.6	54	10.7	6.8
C.L.	R	38.2	28.1	3.4	45	9.6	4.7
D.M.	L	62	14	4.4	52	4	4.6
G.M.	R	19	7	6.8	46	3.9	8.2
C.S.	R	50	12	4.8	50	12	6.5
F.V.	L	56	15	4.9	52	7	5.5
C.Z.	R	49	46	4.5	48	19	5.0

Abbreviations: VCS = sensory conduction velocity (m/s); AMP = sensory nerve action potential amplitude (µV); LDS = sensory distal latency (ms); VCM = motor conduction velocity (m/s); AMP2 = motor action potential amplitude (mV); and LDM = motor distal latency (ms). Abbreviations: R (right) L (left).

**Table 5:** Historical preoperative EMG measurements in Group B (collected prior to surgery, 2016–2021).

Patient	Hand	VCS (m/s)	AMP (µV)	LDS (ms)	VCM (m/s)	AMP2 (mV)	LDM (ms)
B.G.	R	36	20	3.6	50	4.6	6.5
O.M.	R	50	12	4.2	52	8	6.0
O.M.	L	48	10	4.6	51	13	5.0
P.A.	L	60	6	5.4	60	7	5.4
R.R.	R	54.5	4.4	4.1	48	3.9	3.0

Abbreviations: R (right) L (left).

**Table 6:** One-month postoperative ultrasound measurements (Group A).

Patient	Hand	Area at 2 cm Proximal (mm^2^)	Width at 2 cm Proximal (mm)	Thickness at 2 cm Proximal (mm)	Area at Pisiform (mm^2^)	Width at Pisiform (mm)	Thickness at Pisiform (mm)	Thickness at 3 cm Distal (mm)
R.B.	R	15.0	5.3	3.3	7.5	5.5	2.8	2.2
C.C.	L	12.0	6.5	2.8	6.0	5.0	2.1	1.8
M.C.	R	11.5	7.0	1.8	5.0	3.8	2.0	1.6
L.E.D.	L	11.0	3.2	3.0	13.0	7.0	1.9	1.3
G.I.	L	10.5	6.0	2.2	10.0	6.0	4.0	3.5
C.L.	R	9.0	5.4	2.1	13.0	6.5	2.3	1.8
D.M.	L	13.0	5.8	3.5	11.5	5.5	3.1	2.7
G.M.	R	16.0	5.5	3.5	8.0	5.8	2.7	2.3
C.S.	R	9.5	5.8	2.1	11.0	8.5	1.6	1.1
F.V.	L	13.0	5.8	3.4	12.0	5.2	3.0	2.4
C.Z.	R	8.0	4.2	2.6	6.0	4.8	1.7	1.4

Note: CSA is measured only at proximal and pisiform levels. At 3 cm distal, only vertical thickness (in mm) is recorded due to limited reproducibility of CSA at this level. Abbreviations: R (right) L (left).

**Table 7:** Three-month postoperative ultrasound measurements (Group A).

Patient	Hand	Area at 2 cm Proximal (mm^2^)	Width at 2 cm Proximal (mm)	Thickness at 2 cm Proximal (mm)	Area at Pisiform (mm^2^)	Width at Pisiform (mm)	Thickness at Pisiform (mm)	Thickness at 3 cm Distal (mm)
R.B.	R	14.0	5.2	3.1	7.0	5.4	2.7	2.1
C.C.	L	11.5	6.2	2.7	5.8	4.9	2.0	1.7
M.C.	R	11.0	6.8	1.7	4.8	3.7	2.0	1.5
L.E.D.	L	10.5	3.1	2.9	12.5	6.8	1.5	1.2
G.I.	L	10.0	5.8	2.1	9.5	5.8	2.2	1.7
C.L.	R	8.5	5.2	2.0	12.5	6.3	2.1	1.7
D.M.	L	12.5	5.7	3.4	11.0	5.4	2.9	2.6
G.M.	R	15.0	5.4	3.4	7.5	5.7	2.7	2.2
C.S.	R	9.0	5.6	2.0	10.5	8.3	1.4	1.0
F.V.	L	12.5	5.7	3.3	11.5	5.1	2.7	2.3
C.Z.	R	7.8	4.1	2.5	5.8	4.7	1.8	1.3

Note: CSA is measured only at proximal and pisiform levels. At 3 cm distal, only vertical thickness (in mm) is recorded due to limited reproducibility of CSA at this level. Abbreviations: R (right) L (left).

**Table 8:** Six-month postoperative ultrasound measurements (Group A).

Patient	Hand	Area at 2 cm Proximal (mm^2^)	Width at 2 cm Proximal (mm)	Thickness at 2 cm Proximal (mm)	Area at Pisiform (mm^2^)	Width at Pisiform (mm)	Thickness at Pisiform (mm)	Thickness at 3 cm Distal (mm)
R.B.	R	13.5	5.0	3.0	6.5	5.2	2.2	2.0
C.C.	L	12.0	6.0	2.5	5.5	4.8	1.9	1.6
M.C.	R	10.5	6.5	1.6	4.5	3.5	1.6	1.4
L.E.D.	L	6.3	4.6	1.7	7.0	4.8	2.4	1.9
G.I.	L	13.2	6.4	2.6	12.0	6.0	2.1	1.7
C.L.	R	9.0	5.0	2.1	10.0	5.5	1.7	1.5
D.M.	L	17.3	6.5	3.2	18.0	8.4	3.0	2.6
G.M.	R	15.5	6.0	3.0	9.0	6.5	2.5	2.2
C.S.	R	10.5	5.8	2.3	11.0	8.0	1.8	1.3
F.V.	L	11.8	5.5	2.3	10.9	6.1	2.2	2.0
C.Z.	R	8.2	4.4	2.3	7.5	5.0	2.0	1.7

Note: CSA is measured only at proximal and pisiform levels. At 3 cm distal, only vertical thickness (in mm) is recorded due to limited reproducibility of CSA at this level. Abbreviations: R (right) L (left).

**Table 9:** Ultrasound measurements in control group (Group C).

Patient	Hand	Area at 2 cm Proximal (mm^2^)	Width at 2 cm Proximal (mm)	Thickness at 2 cm Proximal (mm)	Area at Pisiform (mm^2^)	Width at Pisiform (mm)	Thickness at Pisiform (mm)	Thickness at 3 cm Distal (mm)
A.G.	L	7.3	5.6	1.8	8.7	6.0	1.5	1.3
B.G. (1)	R	10.6	5.2	2.6	8.0	4.8	1.6	1.3
B.G. (2)	R	10.0	4.6	3.2	9.0	5.0	1.7	1.5
C.G.	L	8.8	5.0	2.8	8.3	5.0	1.7	1.4
C.F.	L	10.0	4.6	3.2	7.6	5.3	1.7	1.4
D.P.C.	L	7.0	3.7	2.2	13.0	7.8	2.0	1.7
D.G.	L	10.0	4.8	2.5	9.0	7.0	1.9	1.5
G.R.	R	10.8	5.2	2.6	9.6	6.4	1.9	1.6
L.D.	R	6.8	4.0	2.3	8.3	6.2	1.7	1.4
O.G.	R	6.5	4.0	2.4	10.9	6.1	1.9	1.5
O.M.	L	7.9	4.6	2.2	10.7	6.7	1.9	1.7
O.C.	R	7.6	6.0	1.8	7.0	5.1	2.0	1.6
S.D.F.	L	9.2	4.8	2.9	13.0	6.2	1.4	1.2
T.M.	R	6.0	4.5	1.7	7.6	6.0	2.0	1.5

Abbreviations: R (right) L (left).

**Table 10:** Comparison of ultrasound parameters among Groups A (preoperative), B (long-term postoperative), and C (baseline healthy controls).

Parameter	Group A (*n* = 11)	Group B (*n* = 6)	Group C (*n* = 14)	*p*-Value (A vs. C)
CSA at 2 cm proximal (mm^2^)	13.0 ± 3.0	10.5 ± 2.0	8.5 ± 1.5	<0.001
Width at 2 cm proximal (mm)	5.5 ± 0.8	5.0 ± 0.7	4.8 ± 0.5	0.002
Thickness at 2 cm proximal (mm)	3.2 ± 0.6	2.8 ± 0.5	2.5 ± 0.4	<0.001

One-way ANOVA with Bonferroni correction is used for group comparisons. Note: Group A = preoperative carpal tunnel syndrome patients; Group B = long-term postoperative patients (operated between 2016 and 2021 and re-evaluated in 2023–2024); and Group C = healthy baseline controls. CSA = cross-sectional area of the median nerve. *p*-values refer to comparisons between Group A and Group C (independent sample *t*-test).

**Table 11:** Changes in ultrasound parameters in Group A over time (repeated measures analysis).

Parameter	Preoperative	1-Month Post-Op	3-Month Post-Op	6-Month Post-Op	*p*-Value (Repeated Measures ANOVA)
CSA at Pisiform (mm^2^)	12.0 ± 3.0	10.0 ± 2.5	9.0 ± 2.0	8.5 ± 1.8	<0.001
Thickness at Pisiform (mm)	2.5 ± 0.5	2.2 ± 0.4	2.0 ± 0.3	1.8 ± 0.3	0.001

Repeated measures ANOVA with post hoc pairwise comparisons is used to assess temporal changes. Significant reductions in CSA at the pisiform level are observed at 1, 3, and 6 months compared to baseline (*p* < 0.05). No statistically significant difference is found between 3 and 6 months (*p* = 0.072). Note: CSA is measured only at proximal and pisiform levels. At 3 cm distal, only vertical thickness (in mm) is recorded due to limited reproducibility of CSA at this level.

## Data Availability

The raw data supporting the conclusions of this article will be made available by the authors upon request.

## Supplementary Materials

The following supporting information can be downloaded at https://www.mdpi.com/article/10.3390/diagnostics15131593/s1, File S1: Standardized Ultrasound Protocol for CTS Evaluation.

## Abbreviations

The following abbreviations are used in this manuscript:

CTS	Carpal tunnel syndrome
EMG	Electromyography
NCS	Nerve conduction studies
CSA	Cross-sectional area
VCS	Sensory conduction velocity (m/s)
AMP	Sensory nerve action potential amplitude (µV)
LDS	Sensory distal latency (ms)
VCM	Motor conduction velocity (m/s)
AMP2	Motor action potential amplitude (mV)
LDM	Motor distal latency (ms)
