# Supplementary material for: Standardized High-Resolution Ultrasound Protocol for the Diagnosis and Monitoring of Carpal Tunnel Syndrome: A Mixed-Design Observational Study
Source: Diagnostics (Basel). 2025 Jun 23;15(13):1593. doi: 10.3390/diagnostics15131593 (PMC12249076; doi:10.3390/diagnostics15131593)
Supplement: Supplementary file 1 [file diagnostics-15-01593-s001.zip › diagnostics-3637141-supplementary.pdf]

Supplementary File – Standardized Ultrasound Protocol for CTS Evaluation

Patient Positioning

- The patient is seated comfortably with the elbow flexed at 90° and the forearm supinated.
- The hand is supported on the examination table in a relaxed position.
- Pre-warmed ultrasound gel is applied to the wrist to improve acoustic coupling and patient comfort.

Probe Orientation and Pressure

- A high-frequency linear probe is used (≥12 MHz).
- The probe is oriented perpendicularly to the longitudinal axis of the forearm to obtain transverse images of the median nerve.
- Minimal transducer pressure is applied to avoid deformation or compression of the nerve.
- Generous ultrasound gel is used to avoid contact artifacts and minimize anisotropy.

Measurement Levels

- Level 1: 2 cm proximal to the pisiform bone (proximal forearm).
- Level 2: At the level of the pisiform bone (carpal tunnel inlet).
- Level 3: 3 cm distal to the pisiform bone (carpal tunnel outlet).
- Measurement points are marked using a flexible ruler along a central longitudinal line drawn on the volar aspect of the wrist and palm.

Measurement Parameters

- At Levels 1 and 2, the cross-sectional area (CSA) of the median nerve is measured by tracing the internal hypoechoic border.
- At Level 3, only the vertical thickness of the median nerve is recorded due to morphological variability and nerve branching.
- Each measurement is repeated three times, and the average is recorded.

Machine Settings

- Ultrasound depth is set between 3 and 4 cm, depending on wrist thickness.
- Gain is adjusted between 55% and 60% to optimize image contrast without oversaturation.
- Focus is placed at the level of the nerve.
- The same ultrasound system and preset configuration are used for all patients.
- Minor adjustments are allowed only to improve clarity without altering measurement consistency.
